# Highly Robust and Multimodal PVA/Aramid Nanofiber/MXene Organogel Sensors for Advanced Human–Machine Interfaces

**DOI:** 10.3390/bios16040229

**Published:** 2026-04-20

**Authors:** Guofan Zeng, Leiting Liao, Zehong Wu, Jinye Chen, Peidi Zhou, Yihan Qiu, Mingcen Weng

**Affiliations:** 1 Department of Physical Education, Fujian University of Technology, Fuzhou 350118, China; 2 Institute of Biology and Chemistry, Fujian University of Technology, Fuzhou 350118, China; 3 School of Smart Marine Science and Technology, Key Laboratory of Marine Smart Equipment, Fujian Province University, Fuzhou 350118, China

**Keywords:** MXene, aramid nanofiber, flexible sensor, strain sensor, pressure sensor, triboelectric nanogenerator

## Abstract

Flexible and wearable electronics require soft sensing materials that balance mechanical compliance, stable signal transduction, and durability for human–machine interfaces (HMIs). To address the limitations of single-filler systems, we propose a poly(vinyl alcohol) (PVA)/aramid nanofiber (ANF)/MXene organogel (PAM) as a multifunctional soft platform. This design integrates a PVA physically crosslinked network with ANF for mechanical reinforcement and MXene for electrical functionality. The optimized PAM composite exhibits outstanding mechanical properties, including a fracture stress of 2931 kPa, a fracture strain of 676%, and a fracture toughness of 9.04 MJ m^−3^. Importantly, PAM serves as a single material platform configurable into three sensing modalities. The resistive strain sensor achieves a gauge factor of 3.1 over 10–100% strain and enables the reliable recognition of human joint movements and gestures. The capacitive pressure sensor delivers a sensitivity of 0.298 kPa^−1^, rapid response/recovery times of 30/10 ms, and is integrated with a wireless module to control a smart car. Furthermore, the PAM-based triboelectric nanogenerator (TENG) delivers excellent electrical outputs (*V*_oc_ = 123 V, *I*_sc_ = 0.52 μA, *Q*_sc_ = 58 nC) and functions as a self-powered smart handwriting pad, achieving a machine-learning-based recognition accuracy of 97.6%. This work demonstrates the immense potential of the PAM organogel for advanced, self-powered HMIs.

## 1. Introduction

Flexible and wearable electronics are increasingly used for continuous physiological monitoring, motion capture, and interactive human–machine interfaces (HMIs), motivating the development of soft sensing materials that can conform to complex, dynamic skin surfaces (e-skin) [1,2,3,4,5]. In practical deployments, these materials must integrate multiple, sometimes competing attributes: mechanical compliance to match large deformations, stable electromechanical signal transduction under repeated loading, and sufficient robustness to tolerate abrasion, sweat exposure, and long-term cycling [6,7,8,9]. For HMI applications in particular, sensors are expected to provide reliable recognition of multimodal inputs such as strain, pressure, and touch, while maintaining low-power operation and system-level compatibility with data acquisition and wireless communication modules [10,11,12]. Achieving this combination of stretchability, sensitivity, and durability remains a core materials challenge.

Among candidate soft conductors and sensing matrices, poly(vinyl alcohol) (PVA)-based gels and organogels have attracted attention because their abundant hydroxyl groups enable dense hydrogen-bonding interactions, facilitating physically crosslinked networks with high deformability and shape adaptability [13,14,15]. PVA gels can be processed using mild routes such as freeze–thaw cycling, which provides a scalable strategy to tune crystallite-assisted junctions and viscoelastic behavior without introducing permanent covalent crosslinkers [16,17,18,19,20]. Moreover, organogel designs that incorporate nonvolatile or low-volatility solvents can mitigate water loss and improve environmental stability, which is important for wearable operation over extended periods [21,22]. However, PVA gel systems still face practical constraints, including limited mechanical reinforcement under high strain, time-dependent creep or stress relaxation, and potential trade-offs between ionic/electronic transport and mechanical integrity in multifunctional devices.

To address the above limitations, conductive and reinforcing fillers are commonly introduced into polymer gels to build percolated transport pathways and enhance load bearing [23,24,25]. Aramid nanofibers (ANFs) provide high aspect ratio reinforcement and strong interfacial interactions, which can improve mechanical strength and suppress creep in physically crosslinked networks, but ANF-dominant systems typically rely on ionic conduction or limited conductive additives, constraining signal levels and multifunctionality [26,27,28]. In contrast, two-dimensional MXenes offer high conductivity and rich surface terminations that facilitate charge transport and interfacial coupling in soft matrices, yet MXene-rich composites may suffer from restacking, oxidation sensitivity, and mechanical brittleness when electrical percolation is prioritized [29,30,31]. Thus, single-filler strategies often exhibit a trade-off between mechanical resilience and electrical performance; to the best of our knowledge, an organogel design that leverages complementary ANF and MXene functions within a PVA matrix for flexible sensing and self-powered HMI remains underexplored [32,33].

Here, we propose a poly(vinyl alcohol) (PVA)/aramid nanofiber (ANF)/MXene organogel (PAM) as a multifunctional soft platform for flexible sensing and self-powered human–machine interfaces. The design integrates a PVA-based physically crosslinked network with ANF-enabled mechanical reinforcement and MXene-enabled electrical functionality, aiming to balance compliance, robustness, and stable signal transduction within a single material system [18,34,35]. A freeze–thaw process is employed to generate crystalline junctions and hydrogen-bonded interactions in the PVA matrix, while promoting intimate interfacial contacts among the components [36]. In parallel, an in situ dispersion strategy is adopted to distribute ANFs and MXene sheets within the organogel phase, suppressing aggregation and supporting the formation of mechanically continuous and electrically connected pathways [18,37]. This dual-network rationale motivates PAM as a tunable matrix for wearable-integrated devices.

Within this framework, three sensing modalities are considered: resistive/piezoresistive sensors typically offer simple structures and straightforward readout, capacitive sensors are often attractive for stable transduction but can be sensitive to geometric spacing and parasitic capacitance, and self-powered triboelectric nanogenerators (TENGs) enable signal generation without an external power supply yet usually require dedicated signal conditioning. Importantly, PAM serves as a single material platform that can be configured into resistive, capacitive, and TENG-based self-powered sensors, which constitutes one highlight of this work. For strain monitoring, the PAM resistive sensor is designed to provide GF = 3.1 over 10–100% strain and remain stable for 1000 cycles, enabling recognition of joint bending (finger, elbow, shoulder, knee, neck, wrist). For pressure-mode interaction, a sensitivity of 0.298 kPa^−1^ and response/recovery of 30/10 ms is targeted and integrated with a microcontroller and Bluetooth to control a mobile car. The PAM-TENG delivers *V*oc 123 V, *I*sc 0.52 μA, and *Q*sc 58 nC and is further used as a handwriting pad with machine-learning-based letter discrimination.

## 2. Materials and Methods

**Materials:** Polyvinyl alcohol (PVA, polymerization degree: 1750 ± 50, CAS No. 9002-89-5) was procured from Shanghai Aladdin Biochemical Technology Co., Ltd. (Shanghai, China). Hydrochloric acid (HCl) and lithium fluoride (LiF) were procured from Shanghai Macklin Biochemical Technology Co., Ltd. (Shanghai, China). Ti_3_AlC_2_ MAX (200 mesh, purity verified by X-ray diffraction, CAS No. 1019635-34-7) phase was procured from Jilin Yiyi Technology Co., Ltd. (Changchun, China). Kevlar pulp was procured from DuPont China Holding Co., Ltd. (Shanghai, China). Dimethyl sulfoxide (DMSO) was procured from Shanghai Titan Scientific Co., Ltd. (Shanghai, China). Potassium hydroxide (KOH) was procured from Sinopharm Chemical Reagent Co., Ltd. (Shanghai, China). Deionized water was obtained from a deionization device.

**Characterizations:** The morphologies and microstructures of the composite organogels were characterized using a scanning electron microscope (SEM, Hitachi SU8010, Hitachi High-Technologies America, Inc., Schaumburg, IL, USA). The molecular compositions and chemical interactions were identified using a Fourier transform infrared spectrometer (FTIR, Nicolet iS50, Thermo Fisher Scientific, Shanghai, China). Fourier transform infrared spectroscopy (FTIR) was performed on a Nicolet iS50 spectrometer using the attenuated total reflection (ATR) mode, which is suitable for the direct test of gel bulk samples. The test wavenumber range was 4000–2500 cm^−1^, the spectral resolution was 4 cm^−1^, and 32 scans were accumulated for each sample to improve the signal-to-noise ratio. Supplementary characterization of freeze-dried gel powder was performed using the KBr pellet method under the same spectral parameters. The rheological behaviors were recorded using a rheometer (HAAKE RS150L, Thermo Fisher Scientific, Karlsruhe, Germany). The mechanical properties were evaluated using an electronic universal testing machine (Instron 5969, Instron (China) Co., Ltd., Shanghai, China) at room temperature (25 ± 2 °C) with a relative humidity of 50 ± 5%. The specimen was cut into a rectangular spline with a size of 0.5 cm (width) × 3 cm (gauge length), and the tensile rate was set to 5 mm/min during the test. At least 5 parallel specimens were tested for each group of samples, and the final results are presented as the average value with standard deviation.

**Test method:** At room temperature, the electrical resistance of PAM organogel and the electromechanical performance of the resistive strain sensor were tested with a Keithley 2400 (Keithley Instruments, LLC, Solon, OH, USA) digital source meter (two-electrode configuration). The rectangular specimen (0.5 cm × 3 cm) was the same as that for the tensile test; copper foil electrodes were bonded with conductive silver paste (cured for 24 h at room temperature) with an effective electrode spacing of 2 cm. Under 1 V constant voltage, real-time current was recorded to calculate resistance and relative resistance change. GF was calculated by GF = (ΔR/R_0_)/ε, with at least 3 parallel samples for reliability. The capacitive performance of the pressure sensor was tested by FDC2214 (Texas Instruments, Dallas, TX, USA)capacitance-to-digital converter coupled with Instron 5969 (Instron, Norwood, MA, USA) universal testing machine (room temperature). Static pressure sensitivity test: loading rate 1 mm/min; cyclic stability test: loading frequency 1 Hz. The electrical output of PAM-based TENG was tested by a programmable linear motor (periodic contact-separation motion) and Keithley 6514 electrometer (recording open-circuit voltage, short-circuit current, and transferred charge, Keithley Instruments, LLC, Solon, OH, USA) at room temperature, with a fixed effective contact area of 2 cm × 2 cm.

**Fabrication of PVA/ANF mixture:** To prepare the PVA solution, 10 g of PVA powder was divided into 5 batches (2 g each) and added to 90 g of DMSO, followed by magnetic stirring at 1200 rpm in a 90 °C water bath to ensure complete dissolution. For the preparation of the high-concentration aramid nanofiber (ANF) dispersion, 2 g of Kevlar (PPTA) fibers, 3 g of KOH, 100 mL of DMSO, and 4 mL of deionized water were mixed and mechanically stirred in a sealed environment at room temperature for 4 h until the solution turned dark black. Subsequently, a specific amount of the ANF dispersion was mixed with the PVA solution (maintaining an ANF mass fraction of 2%) and mechanically stirred at 1200 rpm for 2 h at 80 °C to obtain a viscous, pale yellow PVA/ANF mixture. The ANF has a one-dimensional nanofibrous structure with an average diameter of 20–40 nm, an average length of 2–10 μm, and a narrow, uniform size distribution.

**Fabrication of MXene dispersion:** First, 40 mL of HCl (9 M), 2 g of LiF, and 2 g of Ti_3_AlC_2_ powder were added into a PTFE beaker and stirred at 80 °C for 72 h. The resulting mixture was repeatedly washed with deionized water until the pH reached 7. Then, DMSO was added, and the mixture was ultrasonicated for 1 h, followed by centrifugation at 10,000 rpm for 10 min to collect the precipitate. The precipitate was freeze-dried for 3 days to form a solid, which was subsequently ground into MXene powder. Finally, 1.5 g of the MXene powder was dispersed into 30 mL of DMSO and magnetically stirred at 1200 rpm for 18 h at room temperature to produce a 50 mg/mL MXene dispersion, as shown in Figure S1.

**Fabrication of PAM composite organogels:** The as-prepared MXene dispersion was introduced into the PVA/ANF mixture at various MXene-to-PVA/ANF mass ratios (0.1:100, 0.3:100, 0.5:100, 1:100, 5:100, and 10:100). The mixture was mechanically stirred at 1800 rpm in an 80 °C water bath to ensure homogeneous dispersion. The resulting mixture was then poured into molds and subjected to three freeze–thaw cycles, with each cycle consisting of freezing at −40 °C for 10 h and thawing for 1 h. Finally, the physically cross-linked gels were detached from the molds and immersed in a solvent exchange solution (a mixture of DMSO and deionized water with a DMSO volume fraction of 25%) to obtain the mechanically robust PAM composite organogels. The as-prepared organogels exhibited a smooth, black appearance (Figure S2).

## 3. Results and Discussion

### 3.1. Characterization of PAM

**Figure 1:**
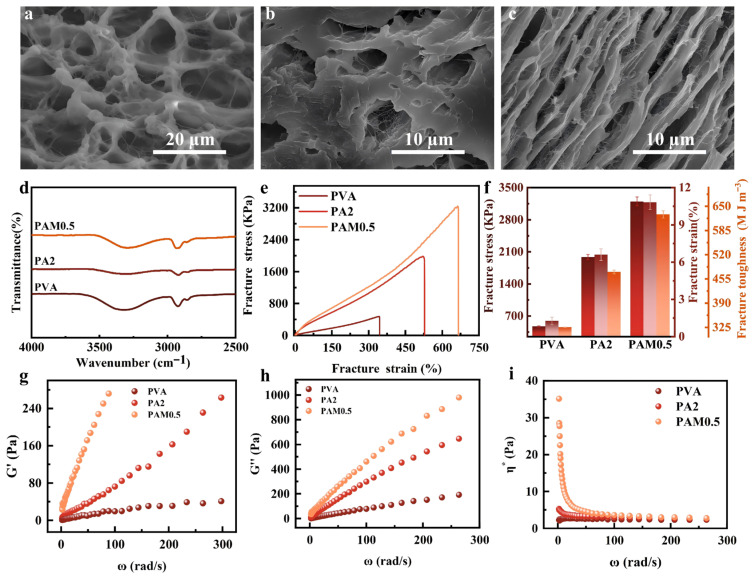
Characterization of PAM composite organogels. SEM of (**a**) PVA, (**b**) PVA-ANF composite organogels, and (**c**) PAM composite organogels. (**d**) FTIR of PVA, PVA-ANF composite organogels, and PAM composite organogels. (**e**) Stress–strain curve of PVA, PVA-ANF composite organogels, and PAM composite organogels. (**f**) Mechanical properties comparison of PVA, PVA-ANF composite organogels, and PAM composite organogels. (**g**) Storage modulus of PVA, PVA-ANF composite organogels, and PAM composite organogels. (**h**) Loss modulus of PVA, PVA-ANF composite organogels, and PAM composite organogels. (**i**) Viscosity of PVA, PVA-ANF composite organogels, and PAM composite organogels.

To investigate the formation causes of the microscopic network structure of the PAM composite organogel and the evolution process of the structural morphology with the cross-linked network, systematic characterizations were conducted. As shown in Figure 1a, it can be clearly identified from the scanning electron microscopy (SEM) image that pure PVA exhibits a relatively loose porous structural feature with thin pore walls, and its pore size distribution shows irregularity, indicating that the pure PVA network is difficult to construct a dense skeleton after the freeze-drying process [38,39]. With the intervention of 2 wt% ANF, the gel pore walls are significantly thickened, and the pore size becomes smaller, resulting in a more regular and orderly pore morphology. More importantly, during this process, numerous elongated ANF fibers are observed embedded or scattered inside the network structure (Figure 1b) [3,4]. This reflects that due to the easy formation of van der Waals forces between 1D ANFs and the hydrogen bond cross-linking interaction with the PVA network, it not only effectively reduces the number of pores and shrinks the pore size but also effectively promotes the improvement of the overall strength of the gel [5]. When 0.5% MXene is further added to the PVA/ANF system (Figure 1c and Figure S3a,b), its pore outline exhibits a smoother and tighter characteristic compared to the former two, and lamellar and fibrous arrangement features can be observed simultaneously [40,41]. It is speculated that this is because the 2D MXene nanosheets and ANF exerted a synergistic effect, constructing a more robust three-dimensional network structure, enhancing the compactness of the internal structure, and providing more dense connection nodes for the formation of conductive channels within the gel. The synergistic modification effect produced by the introduction of ANFs and MXene nanosheets can significantly improve the pore morphology and network uniformity of the PVA-based gel, thereby laying a solid microstructural foundation for subsequent improvements in mechanical and electrical properties. Next, Fourier transform infrared spectroscopy (FTIR) reveals that the broad O-H stretching vibration band of pure PVA at 3320 cm^−1^ changes significantly with the addition of ANF and MXene (Figure S4a) [42,43,44]. The absorption peak redshifts to 3311 cm^−1^ after adding ANF (PA2) and undergoes a further massive redshift to 3292 cm^−1^ while becoming broader and shallower upon introducing 0.5% MXene (Figure 1d and Figure S4b) [45,46]. This pronounced redshift confirms the reconstruction and formation of a stronger, denser new hydrogen-bonded network among the hydroxyl groups of PVA, the amide groups of ANF, and the polar surface functional groups of MXene. These molecular-level structural changes reveal the nanoscale synergistic modification mechanism, providing a solid chemical basis for the significant enhancement in the macroscopic mechanical and rheological properties of the composites. To further corroborate the formation of the new multiple hydrogen-bonded networks indicated by the FTIR analysis, we systematically evaluated the mechanical properties of the composite organogels. As shown in the figures, the mechanical properties of the PA(PVA-ANF) composites display a volcano-shaped trend with increasing ANF content. The PA2(PVA-2wt%ANF) sample achieves the optimal comprehensive mechanical performance, with a maximum fracture strain of 570% (1.66 times that of pure PVA at 344%) and a peak fracture toughness of 5.83 MJ m^−3^. This improvement is attributed to the dense hydrogen-bonding network between ANF and PVA, which facilitates highly efficient energy dissipation during stretching. Consequently, building upon the PA2 formulation, varying concentrations of MXene (0 wt% to 10 wt%) were introduced to investigate the synergistic reinforcement effect on the crosslinked network. As depicted in the stress–strain curves and the corresponding bar charts, the tensile strength and extensibility of the PAM organogels also exhibit an initial increase followed by a decrease as the MXene doping level rises. At low concentrations (0.3–0.5%), the tensile curves elevate significantly, indicating enhanced global mechanical performance. Conversely, excessive MXene content (≥5%) likely causes the agglomeration of nanosheets, leading to stress concentration and heterogeneous interfacial dispersion, which consequently degrades both fracture strain and toughness; thus, it was selected as the foundational matrix for subsequent experiments. Notably, the PAM0.5(PVA-ANF-0.5wt%MXene) composite demonstrates the most outstanding mechanical performance. Its fracture stress reaches 2931 kPa, with an impressive fracture strain of 676%. Furthermore, its calculated fracture toughness is drastically improved to 9.04 MJ m^−3^, significantly outperforming both the undoped and heavily doped groups. These results indicate that an appropriate amount of MXene nanosheets can uniformly disperse within the PVA-ANF network, forming multiple hydrogen bonds and physical crosslinking points that effectively enhance stress transfer and energy dissipation. Given its optimal mechanical robustness and high stretchability—which are highly desirable for flexible electronics—the PAM0.5 composite was selected as the standard material for the subsequent sensor fabrication and performance evaluations.

To further clarify the advancement of the mechanical properties of the PAM composite organogel prepared in this work, we systematically compared its key mechanical parameters with those of previously reported PVA-based composite gel materials in related studies. As shown in the work of Zeng et al., the CNT-PVA binary composite hydrogel modified by a single conductive filler only achieved a fracture stress of 520 kPa, a fracture strain of 180%, and a fracture toughness of 0.51 MJ m^−3^. In contrast, the PAM organogel in this work exhibits a fracture stress of 2931 kPa, a fracture strain of 676%, and a fracture toughness of 9.04 MJ m^−3^, which are 4.6 times, 2.7 times, and nearly 17 times higher than the above binary system, respectively. For the PVA-based PNP hydrogel developed by Zhou et al., the modification strategy mainly focused on the in situ polymerization of conductive polyaniline to meet the functional requirements of underwater energy storage and sensing, without a targeted multi-dimensional synergistic design for the mechanical reinforcement of the PVA matrix. Different from the above single-function modification strategy, this work introduces one-dimensional ANF as the mechanical reinforcement phase and two-dimensional MXene as the functional conductive phase into the PVA matrix at the same time. The dense multi-hydrogen bond cross-linking network constructed by the synergistic interaction of the three components not only achieves a simultaneous leap in tensile strength, stretchability, and fracture toughness, but also effectively balances the mechanical robustness and electrical conductivity of the gel system, which fundamentally solves the long-standing trade-off between mechanical properties and functional properties in traditional single-filler modified PVA gel systems. This dual-nanofiller synergistic reinforcement strategy also provides a universal design idea for the preparation of high-performance multifunctional PVA-based gel materials for flexible sensing [13,14].

Finally, dynamic rheological tests further evaluated the network stability of the composite gel. With the sequential addition of ANF and MXene, both the storage modulus (G′) and loss modulus (G″) of the system increase substantially, and G′ is consistently and significantly higher than G″, indicating the formation of a highly elastic solid-like network structure within the system. Simultaneously, in steady-state shear tests, the PAM0.5% gel exhibits typical pseudoplastic fluid (shear-thinning) characteristics, featuring high initial viscosity at low shear rates and a rapid decrease in viscosity at high shear rates. This rheological property not only confirms the highly uniform dispersion of MXene within the network but also endows the composite system with excellent coating and film-forming processability. Based on the perfect synergy of the dense morphology, multiple hydrogen bond reconstructions, ultra-high mechanical toughness, and excellent processability.

### 3.2. Performances and Applications of PAM-Based Strain Sensor

**Figure 2:**
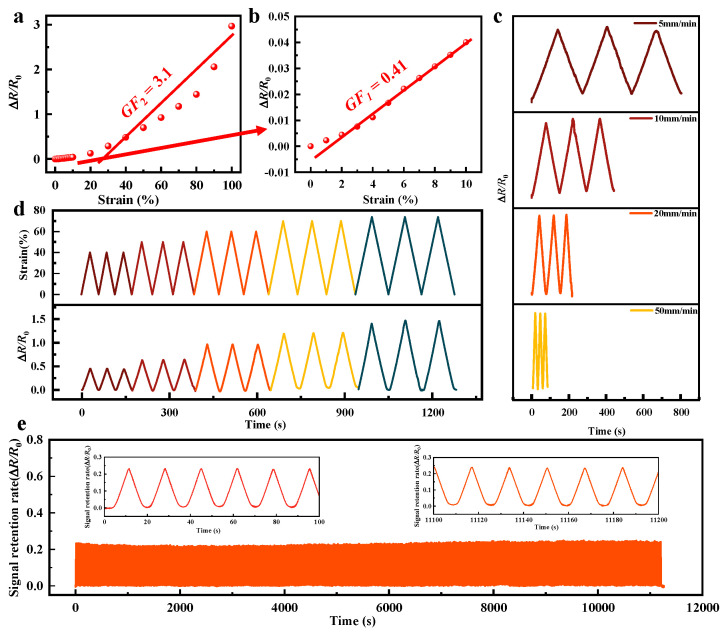
Performance of PAM-based strain sensor. (**a**,**b**) GF of PAM-based strain sensor. (**c**) Rate of resistance change of PAM-based strain sensor at different strain rates. (**d**) Resistance change corresponding to 40–80% strain of PAM-based strain sensor. (**e**) Stability curve of PAM-based strain sensor.

The strain-sensing mechanism of the PAM organogel is fundamentally based on the piezoresistive effect, where the 3D flexible skeleton constructed by PVA and ANF via non-covalent interactions supports a stable conductive MXene percolation network [47,48]. During external stretching, the deformation of the polymer matrix forces the sliding and partial separation of MXene nanosheets, which disrupts the network connectivity and lengthens the electron transport pathways, leading to a significant increase in macroscopic resistance [49,50]. Consequently, the sensor exhibits a highly linear strain-resistance relationship at low strains, while at higher strains, the extensive rupture and dynamic reconfiguration of the MXene network cause a more pronounced variation in resistance, endowing the PAM material with exceptional strain sensitivity. The experimental results fully validate this wide-range sensing capability. The PAM gel exhibits a sensitive response to minute deformations within the strain range below 10%, achieving a sensitivity factor (GF) of 0.41. Within the broader strain range of 10% to 100%, the electrical response becomes significantly more pronounced, with the GF value surging to 3.1 (Figure 2a,b). This outcome demonstrates that PAM-composite organic gels exhibit exceptional sensitivity and stability under high-strain conditions, revealing immense application potential in large-deformation monitoring fields such as human motion capture or deformation detection in flexible mechanical components. This typical segmented sensitivity variation (i.e., two distinct GF values) is primarily attributed to the dynamic microstructural evolution of the MXene conductive network within the PAM organogel at different strain stages. In the low-strain region (<10%), the deformation of the flexible PVA-ANF skeleton is relatively mild, and the attached MXene nanosheets mainly undergo relative sliding. During this stage, although the reduced overlapping area between nanosheets increases contact resistance, electrons can still maintain cross-layer transport through localized physical contact or the quantum tunneling effect. This preserves a high overall integrity of the conductive network, resulting in a gradual and highly linear change in resistance. However, as the strain exceeds 10% and enters the high-strain region, the excessive stretching of the polymer matrix causes the relative displacement between MXene nanosheets to breach the physical overlapping limit, leading to large-scale sliding and complete separation. At this point, continuous electron transport pathways are massively severed, and the distance between adjacent nanosheets increases sharply, exceeding the critical threshold for effective tunneling. This process is even accompanied by the initiation and rapid propagation of microcracks within the network. Such an “avalanche-like” rupture and dynamic reconfiguration of the conductive network causes the macroscopic resistance of the system to increase drastically, thereby endowing the sensor with a significantly surged, high sensitivity over a broad strain range. As shown in Figure 2c evaluates the dynamic response characteristics of the organogel sensor under varying stretching rates (from 5 to 50 mm/min). The results demonstrate that despite the broad range of strain rates, the waveforms and peak values of the relative resistance change (Δ*R*/*R*_0_) remain highly consistent, exhibiting excellent strain-rate independence. This stable response, entirely unaffected by the loading speed, confirms that the internal conductive network can achieve rapid and synchronized reversible reconfiguration under various deformation rates, thereby ensuring its reliability for high-fidelity monitoring of complex human motions with variable speeds in practical applications. Furthermore, as depicted in Figure 2d, to further evaluate the dynamic sensing performance, the organogel was subjected to step-wise cyclic tensile testing under various strain amplitudes (40% to 80%), exhibiting a highly synchronized and stable stepped resistance response. After multiple loading-unloading cycles at each fixed strain, the peak resistance signals remained highly consistent and rapidly recovered to the initial baseline upon complete unloading, demonstrating negligible hysteresis. Finally, to verify the reliability of the sensor in practical high-frequency working environments, Figure 2e comprehensively evaluates the sensing stability of the PAM organogel under long-term continuous mechanical loading. Over the course of a stretch-release cyclic test comprising more than 1000 cycles (lasting over 11,000 s), the Δ*R*/*R*_0_ of the sensor maintained exceptional uniformity, with no discernible baseline drift or signal degradation observed. Notably, as depicted in the locally magnified insets at the initial and final stages of the test, the waveform and peak characteristics of the resistance response remain nearly perfectly coincident with the initial cycles, even after prolonged high-frequency cycling. This outstanding anti-fatigue property and long-term mechanical stability are primarily attributed to the effective synergy between the flexible PVA matrix and the reinforcement of ANFs, which successfully buffers stress concentration and prevents irreversible damage to the MXene conductive network. These robust testing results fully highlight the extraordinary durability of the organogel, providing compelling data support for its long-term practical applications in continuous, high-frequency monitoring fields such as wearable electronics and flexible robotics.

**Figure 3:**
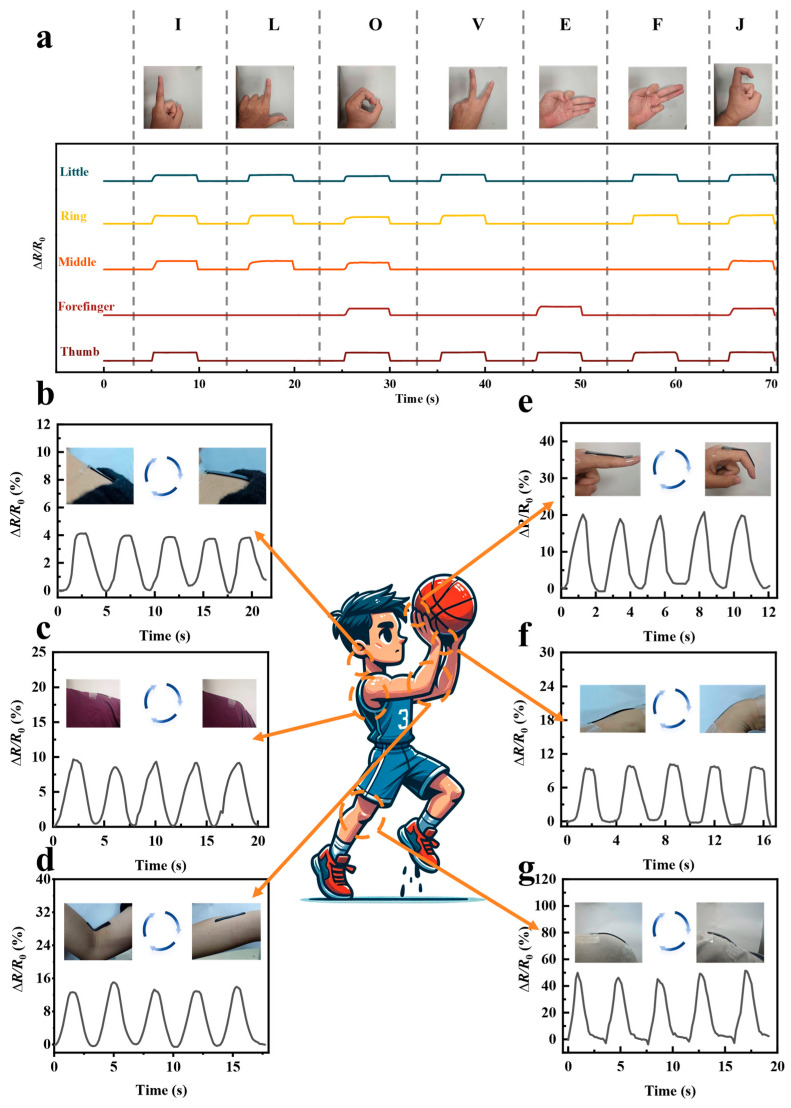
Applications of PAM-based strain sensor. (**a**) The rate of change in resistance for the sensor attached to each finger representing the gestures for “I,” “L,” “O,” “V,” “E,” “F,” and “J.” (**b**–**g**) Applications of sensors on different parts of the body.

To further explore the potential of this sensor in fine and complex interactions, a multi-channel gesture recognition experiment was designed (Figure 3a). Five independent sensors were attached to the joints of the tester’s five fingers to record the waveform characteristics while continuously spelling the phrase “I love FJ”. By accurately capturing and synergistically analyzing the five-channel signals, the motion signature of each letter can be clearly decoded. For instance, when forming the letter “I”, the forefinger remains straight without bending (its corresponding Δ*R*/*R*_0_ curve remains flat), while the thumb, middle, ring, and little fingers are bent, generating distinct peak signals on their respective curves that clearly capture the strain changes caused by the bending actions. Similarly, when expressing the letter “L”, the thumb and forefinger remain straight (their corresponding sensors detect no obvious waveform fluctuations), while the middle, ring, and little fingers are bent, prompting their sensors to generate corresponding peak features, reflecting their sensitive response to deformation. The gesture variations of subsequent letters (e.g., “o”, “v”, “e”) were equally translated into unique waveform combinations by the multi-channel system. By analyzing the signal characteristics of each gesture one by one, we systematically verified the composite sensor’s precise perception capability for multi-dimensional fine movements. To evaluate the practical strain-sensing potential of the dumbbell-shaped PAM composite organogel sensor, we attached it to multiple key human joints to monitor multiscale motion-induced deformations. Benefiting from its high sensitivity and broad response range, the sensor can precisely record Δ*R*/*R*_0_ during the periodic bending and extension of various body parts. Specifically, for subtle to moderate movements such as the neck (Figure 3b) and wrist (Figure 3f), the sensor outputted stable resistance change signals of approximately 4% and 10%, respectively, featuring smooth waveforms and high response consistency. When monitoring large-angle joint bending at the fingers (Figure 3c), lower back (Figure 3c), elbows (Figure 3d), and knees (Figure 3g), the signal amplitude increased significantly; in particular, the deep bending of the knee generated a prominent peak of nearly 50%. The waveforms from all tested channels exhibited sharp peak-and-valley features and excellent repeatability, fully validating the high resolution and reliability of the sensor in sports biomechanics monitoring and rehabilitation medicine.

### 3.3. Performances and Applications of PAM-Based Pressure Sensor

**Figure 4:**
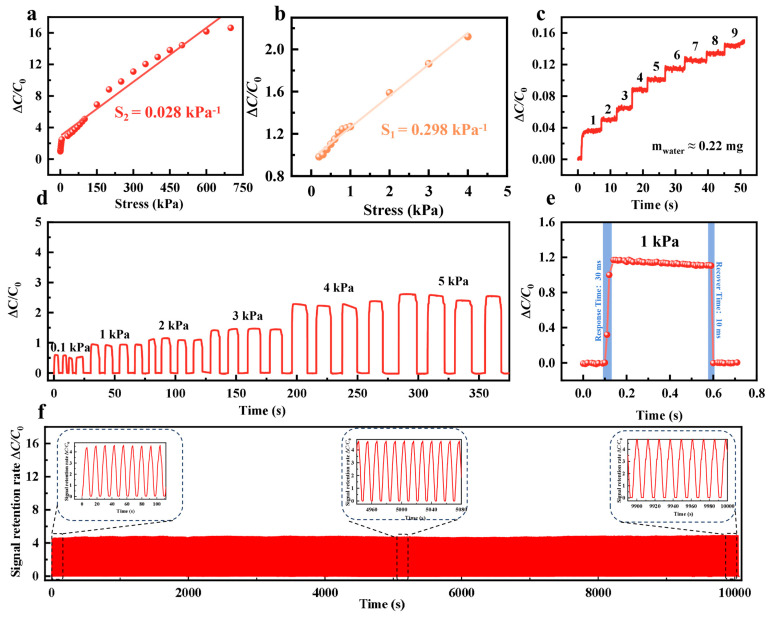
Performance of PAM-based pressure sensor. (**a**,**b**) Relative capacitance rate of change curves of the PAM-based pressure sensor at different pressures. (**c**) Capacitance change rate of PAM-based pressure sensors with different water droplet volumes. (**d**) Capacitance change rate curve under different pressure cycles of PAM-based pressure sensor. (**e**) Response/recovery time of PAM-based pressure sensor. (**f**) Stability curve of PAM-based pressure sensor.

Generally, resistive sensors offer advantages such as structural simplicity, straightforward signal readout, and broad measurement ranges; however, they often face limitations regarding long-term stability, hysteresis, and susceptibility to environmental temperature and humidity. In contrast, although capacitive pressure sensors require more sophisticated readout circuitry, they are highly favored in flexible tactile sensing due to their ultra-low static power consumption, excellent dynamic response capabilities, and ultra-high sensitivity to minute forces [49,51,52]. Building on this, we further constructed a capacitive pressure sensor with a sandwich architecture, utilizing the PAM composite organogel as the core dielectric layer encapsulated by two copper foil electrodes. Distinct from the aforementioned piezoresistive mechanism relying on the disruption of conductive networks, the capacitive sensing mechanism of this device is primarily governed by deformation-induced variations in electrode spacing and effective dielectric constant. When external pressure is applied to the sensor surface, the 3D flexible network constructed by PVA and ANF undergoes compressive deformation, directly reducing the macroscopic distance between the top and bottom copper electrodes. More crucially, the highly conductive 2D MXene nanosheets uniformly dispersed within the matrix act as an abundance of “micro-capacitors” before reaching the percolation threshold. Under compression, these adjacent, non-contacting MXene sheets are forced closer together, significantly enhancing the internal interfacial polarization effect and thereby dramatically increasing the effective dielectric constant of the dielectric layer. The synergistic effect of the reduced macroscopic electrode spacing and the sharply increased microscopic effective dielectric constant empowers the PAM composite sensor to efficiently and accurately translate minute external pressures into pronounced capacitance signal changes. A schematic of the piezoresistive pressure sensor based on the PVM hydrogel is shown in Figure S7a. As shown in Figure 4a,b, the sensor exhibits a high sensitivity of 0.298 kPa^−1^ in the low-pressure regime (<4 kPa); once the applied pressure exceeds 4 kPa and complete electrode-gel contact is achieved, bulk compression dominates, and the sensitivity smoothly transitions to 0.028 kPa^−1^. Ultimately, the introduction of MXene constructs an abundance of “micro-capacitors” within the matrix to significantly boost the interfacial polarization effect, endowing the composite organogel with acute and stable capacitive sensing performance over a broad pressure range. As shown in Figure 4c, to further evaluate the limit of detection (LOD) of the sensor, its dynamic capacitance response to the continuous dripping of minuscule water droplets (weighing only ~0.22 mg each) was tested. With the successive accumulation of nine individual droplets, the relative capacitance signal exhibited an exceptionally clear and stable step-wise increase, accurately capturing every ultra-light mass loading event. This outstanding stepped response not only verifies the sensor’s ultra-low LOD and superior signal resolution but also highlights its immense application potential in micro-pressure monitoring, such as detecting subtle airflows, insect crawling, or human pulses. As illustrated in Figure 4d, to evaluate the dynamic sensing stability of the sensor over a broad measurement range, stepped cyclic pressures from 1 kPa to 5 kPa were applied (with five repeated loading–unloading cycles at each specific pressure). The test results demonstrate that the relative capacitance change signal (ΔC/*C*_0_) not only accurately distinguishes external pressures of various magnitudes but also maintains highly consistent waveform peaks and rapid baseline recovery capabilities during multiple cycles under identical pressure conditions. This exceptional repeatability and hysteresis-free response exhibited across a wide dynamic pressure range provide a reliable guarantee for its practical applications in smart wearable devices and complex, high-sensitivity dynamic perception scenarios. Upon applying a pressure of 1 kPa to the PAM composite gel sensor, the device exhibited rapid response and recovery times of 30 ms and 10 ms, respectively, highlighting its superior dynamic sensing performance (Figure 4e). As shown in Figure 4f, after undergoing 1200 continuous loading-unloading cycles under a relatively high pressure of 50 kPa, the capacitance response peaks of the sensor exhibited negligible degradation. The magnified insets of the initial and final stages further confirm its exceptional anti-fatigue properties and long-term operational stability. In summary, owing to its outstanding sensitivity, ultra-low limit of detection, and high reliability over a broad measurement range, this PAM composite capacitive pressure sensor demonstrates immense practical application potential in flexible sensing fields such as smart wearables, electronic skins, and microfluidic monitoring.

**Figure 5:**
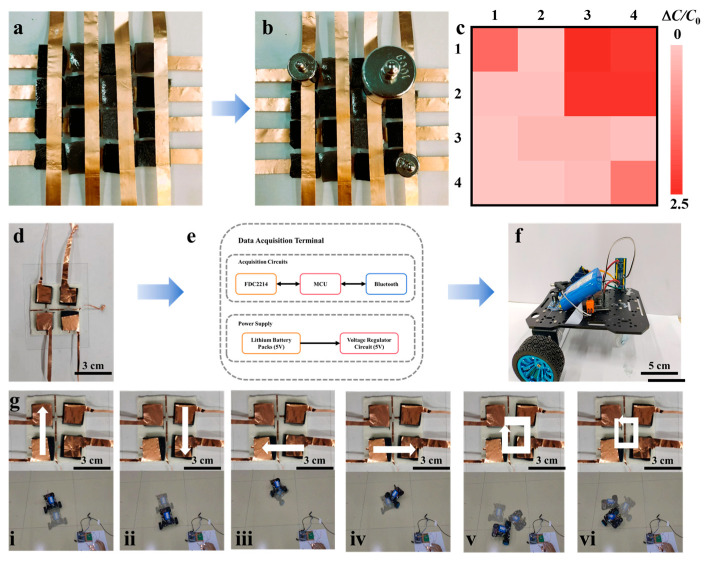
Applications of PAM-based pressure sensor. (**a**,**b**) Optical photograph of PAM-based pressure sensor array. (**c**) Capacitance response heatmap of PAM-based pressure sensor array. (**d**–**f**) Mechanism diagram of intelligent car control. (**g**) Demonstration of the human–machine interface for controlling an intelligent cart using a 2 × 2 sensor array.

Encouraged by these remarkable single-point sensing metrics—including outstanding sensitivity, ultra-low LOD, and broad-range reliability—we sought to demonstrate the PAM organogel’s utility in macroscopic, spatially resolved human–machine interfaces. To evaluate the spatial resolution capability of the sensor, a 4 × 4 pixel array was constructed (Figure 5a), and weights of different masses were applied at various positions (Figure 5b, i.e., 100 g in the top right, 20 g in the top left, and 10 g on the bottom right). The relative capacitance change of each unit was recorded and mapped into a 2D heatmap (Figure 5c). The results indicate that the highest-pressure area (100 g) exhibits the strongest response signal (dark red), followed by the 20 g area, while the lightest load area (10 g) shows the weakest signal, with negligible signal crosstalk in non-pressed regions. This demonstrates the array’s ability to precisely quantify and localize spatially distributed discrete pressures, serving as an efficient and high-precision sensing platform.

Based on these excellent array sensing characteristics, a wireless control system based on a 2 × 2 sensor array was further developed for a human–machine interaction demonstration using a smart car (Figure 5d). As illustrated in Figure 5e, the customized terminal utilizes an FDC2214 capacitance-to-digital converter as the core, combined with a microcontroller (MCU) and a Bluetooth module for signal acquisition and wireless transmission. When a user performs directional sliding presses (e.g., “↑”, “↓”, “←”, “→”, corresponding to Figure 5(gi): forward, Figure 5(gii): reverse, Figure 5(giii): left turn, and Figure 5(giv): right turn) or circular sliding operations (Figure 5(gv,gvi)) on the 2 × 2 array (Figure 5g), specific spatiotemporal capacitance signals are generated. The MCU decodes these feature signals and drives the smart car via Bluetooth to move forward, backward, turn, or rotate in place (Figure 5f,g). This demonstration not only validates the system’s reliability in complex command recognition and wireless communication but also fully proves the immense engineering potential of the PAM composite sensor in advanced human–machine interfaces.

### 3.4. Performances and Applications of PAM-Based TENG

**Figure 6:**
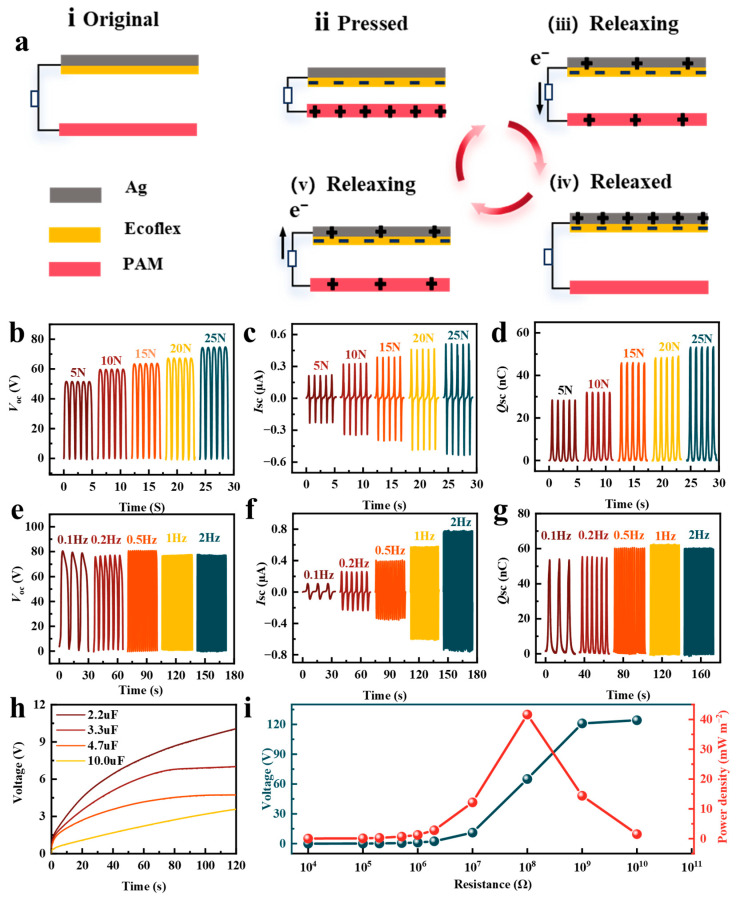
Performances of PAM-based TENG. (**a**) Schematic image of the working mechanism of the PAM-based TENG. (**b**) *V*oc, (**c**) *I*sc, and (**d**) *Q*sc under different impact forces. (**e**) *V*oc, (**f**) *I*sc, and (**g**) *Q*sc under different impact frequencies. (**h**) Charging of the various capacitors with the PAM-based TENG. (**i**) Output power density and voltage of the PAM-based TENG.

Owing to its excellent electrical conductivity, mechanical flexibility, and tunable surface chemistry, the PVA/ANF/MXene (PAM) composite organogel serves not only as a remarkable sensor but also as an ideal triboelectric layer and electrode for flexible triboelectric nanogenerators (TENGs). As illustrated in Figure 6a and Figure S7b, a vertical contact-separation mode TENG was constructed utilizing Ag/Ecoflex as the top triboelectric counterpart and the PAM gel as the bottom active layer. The power generation mechanism relies on the coupling of triboelectrification and electrostatic induction. During the initial pressing state (Figure 6(aii)), the Ecoflex layer comes into intimate contact with the PAM layer. Driven by the difference in electron affinities, electrons transfer from the PAM surface to the Ecoflex surface, leaving the Ecoflex negatively charged and the PAM positively charged. When the external force is removed, the elastic recovery of the materials separates the two layers (Figure 6(aiii)), breaking the electrostatic equilibrium. This separation generates a potential difference, driving electrons to flow from the top Ag electrode to the bottom PAM electrode through the external circuit. Conversely, when the device is compressed again (Figure 6(ai–av)), the separation distance decreases, and electrons flow back, thereby producing a continuous, periodic alternating current. The output performance of the TENG is highly dependent on external mechanical stimuli [53,54,55]. As shown in Figure 6b–d, increasing the applied force from 5 N to 25 N significantly enhances the effective contact area and intimacy between the triboelectric layers, leading to a steady increase in electrical outputs. Under a force of 25 N, the open-circuit voltage (*V*_oc_), short-circuit current (*I*_sc_), and transferred charge (*Q*_sc_) reach approximately 80 V, 0.52 μA, and 55 nC, respectively. Furthermore, the dynamic response to varying triggering frequencies (0.1 Hz to 2 Hz) under a constant force was investigated (Figure 6e–g). Because the maximum separation distance and contact area remain constant, both *V*_oc_ and *Q*_sc_ exhibit remarkable stability across the tested frequencies. In contrast, the *I*_sc_ shows a pronounced positive correlation with frequency—reaching a peak of approximately 0.8 μA at 2 Hz—since higher frequencies accelerate the charge transfer rate (*I* = *dQ*/*dt*). To evaluate its viability as a practical power source, the charging capabilities of the TENG for commercial capacitors (2.2 μF to 10 μF) were tested (Figure 6h). Within ~120 s, the capacitors can be rapidly charged to approximately 10.0 V, 7.0 V, 4.7 V, and 3.5 V, respectively. Additionally, the impedance matching behavior was systematically investigated (Figure 6i). With increasing external load resistance, the output voltage gradually rises and approaches saturation (~120 V near 10^10^ Ω), while the output power density peaks at an optimal matched resistance of 10^8^ Ω, reaching approximately 40 mW m^−2^. These results compellingly demonstrate the immense potential of the PAM-based TENG for mechanical energy harvesting and powering low-consumption electronics.

**Figure 7:**
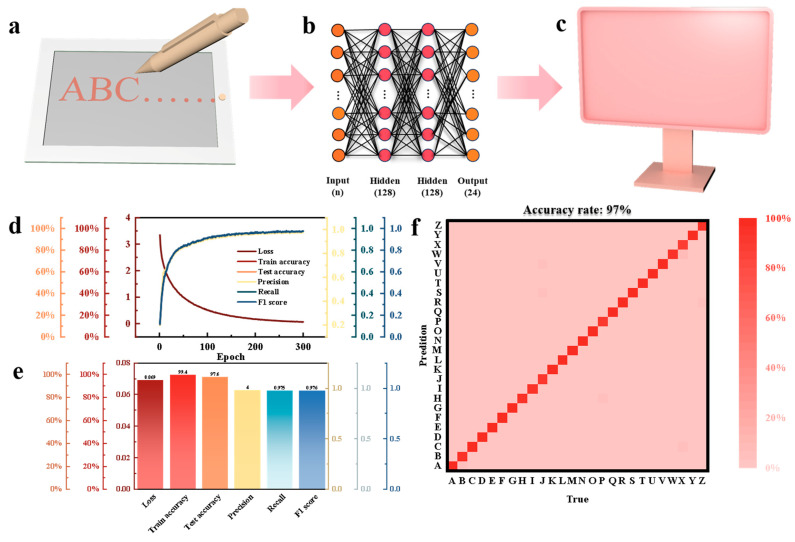
Applications of PAM-based TENG. (**a**–**c**) Schematic diagram of the operating mechanism for an intelligent handwriting pad based on PAM-based TENG. (**d**) Machine learning training process diagram. (**e**) Machine learning training results. (**f**) Smart handwriting pad confusion matrix.

We constructed a self-powered smart handwriting recognition system based on the PAM composite organogel TENG (Figure 7a). When localized dynamic pressure is applied to the writing tablet, the time-varying contact-separation process triggers a characteristic electrical signal. In the writing scenario, the writing actions of different English letters induce the TENG to generate signals with unique waveform characteristics by applying dynamic pressure patterns with temporal differences and spatial distributions. By capturing these signals, we constructed a multi-layer perceptron (MLP) neural network classification model, featuring two hidden layers with 128 neurons each, for real-time pattern recognition (Figure 7b,c). A systematic experimental dataset was constructed for the 26 English letters from ‘A’ to ‘Z’. Based on a supervised learning framework, after 300 iterations (epochs) of training, the MLP model shows excellent performance: the loss function rapidly converges to near zero, while the training accuracy is significantly improved (Figure 7d). Finally, we performed a prediction evaluation for the test set, which showed an outstanding test accuracy of 97.6%, accompanied by exceptionally high precision, recall, and F1 scores (Figure 7e). The corresponding learning curves and the confusion matrix, which demonstrates an overall accuracy rate of 97%, are shown in Figure 7e, Figure 7f, and Figure S8, respectively.

## 4. Conclusions

In summary, this work developed a high-performance poly(vinyl alcohol)/aramid nanofiber/MXene (PAM) composite organogel through a facile freeze–thaw processing and in situ dispersion strategy. Benefiting from the synergistic multiple hydrogen bonding interactions among the three components, a dense and stable physically crosslinked network was successfully constructed within the organogel, which effectively overcomes the long-standing trade-off between mechanical flexibility and electrical conductivity in traditional single-filler gel systems. The optimized PAM organogel achieves an excellent balance of mechanical robustness, stretchability, and stable electrical conductivity, providing a versatile and reliable monomaterial platform for the development of multifunctional flexible electronics.

Based on this PAM organogel platform, we successfully fabricated three types of functional devices with distinct working mechanisms: a resistive strain sensor, a sandwich-structured capacitive pressure sensor, and a vertical contact-separation mode triboelectric nanogenerator (TENG). All devices exhibit outstanding working performance and operational stability, and have been validated in practical application scenarios including multi-scale human motion monitoring, wireless human–machine control of smart cars, and self-powered smart handwriting recognition. In particular, the combination of the TENG-based handwriting pad with a multilayer perceptron machine learning model enables high-precision recognition of 26 English letters, fully demonstrating the application potential of this material system in complex intelligent interaction scenarios.

The key innovative elements of this study are summarized as follows. First, a dual-nanofiller synergistic modification strategy is proposed for PVA-based organogels, where one-dimensional ANF serves as the mechanical reinforcement phase and two-dimensional MXene acts as the functional conductive phase. This design realizes the simultaneous optimization of mechanical and electrical properties of the gel matrix through non-covalent interactions, without the need for complex chemical crosslinking processes. Second, this work realizes the configurable expansion of a single material system to three different sensing/energy harvesting modalities, which simplifies the material design of multifunctional flexible devices and provides a universal material solution for the integrated development of multi-mode wearable electronics. Third, we establish a complete application chain from material microstructure design, macro performance optimization to system-level intelligent human–machine interaction, which lays a solid material and engineering foundation for the development of next-generation flexible electronic skins, wearable health monitoring systems, and advanced self-powered human–machine interfaces.

## Data Availability

The original contributions presented in this study are included in the article/Supplementary Material. Further inquiries can be directed to the corresponding authors.

## Supplementary Materials

The following supporting information can be downloaded at: https://www.mdpi.com/article/10.3390/bios16040229/s1, Figure S1: Uniform distribution of PAM in deionized water; Figure S2: Optical image of PAM gel; Figure S3: (a,b) SEM of PAM composite organogels local magnified view; Figure S4: (a) FTIR of PA5, PA4, PA3, PA2, PA1 and PVA; (b) FTIR of PAM10, PAM5, PAM1, PAM0.5, PAM0.3 and PAM0.1; Figure S5: (a) Mechanical properties comparison of PA5, PA4, PA3, PA2, PA1 and PVA; (b) Mechanical properties comparison of PAM10, PAM5, PAM1, PAM0.5, PAM0.3 and PAM0.1; Figure S6: Stability curve of PAM-based pressure sensor; Figure S7: Supply electrode thickness and connection mode in (a) pressure sensor and (b) TENG structural schematics; Figure S8: Confusion Matrix of MLP-Based Alphabet Recognition with Labeled Precision, Recall and Overall Classification Accuracy.
