# Supplementary material for: Highly Robust and Multimodal PVA/Aramid Nanofiber/MXene Organogel Sensors for Advanced Human–Machine Interfaces
Source: Biosensors (Basel). 2026 Apr 20;16(4):229. doi: 10.3390/bios16040229 (PMC13115146; doi:10.3390/bios16040229)
Supplement: Supplementary file 1 [file biosensors-16-00229-s001.zip › biosensors-4218290-supplementary.pdf]

biosensors

*Supplementary materials*

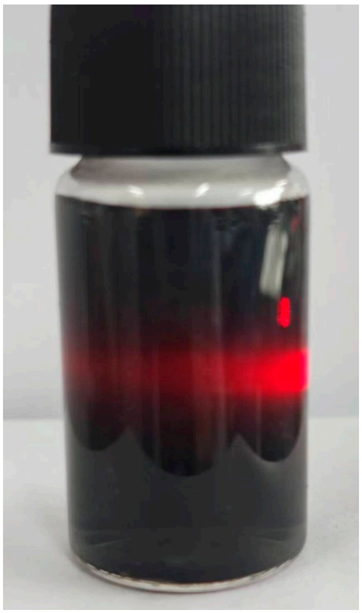

**Figure S1.** Uniform distribution of PAM in deionized water.

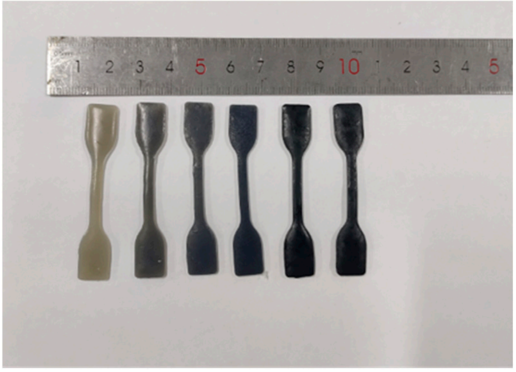


**Figure S2.** Optical image of PAM gel.

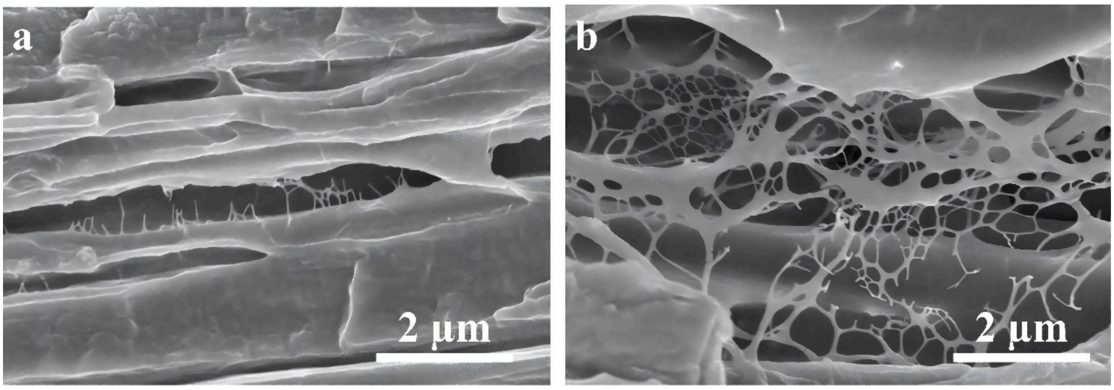


**Figure S3.** (**a, b**) SEM of PAM composite organogels local magnified view.

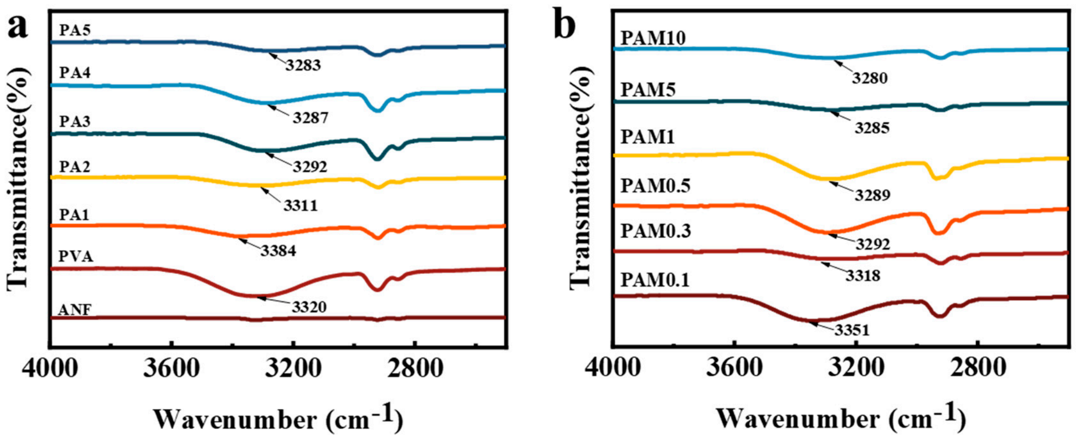


**Figure S4.** (**a**)FTIR of PA5、PA4、PA3、PA2、PA1 and PVA. (**b**) FTIR of PAM10、PAM5、PAM1、PAM0.5、PAM0.3 and PAM0.1.

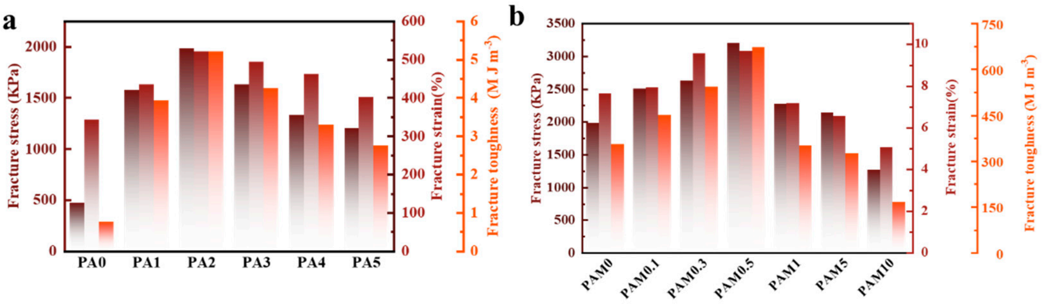


**Figure S5.** (**a**) Mechanical properties comparison of PA5、PA4、PA3、PA2、PA1 and PVA. (**b**) Mechanical properties comparison of PAM10、PAM5、PAM1、PAM0.5、PAM0.3 and PAM0.1.

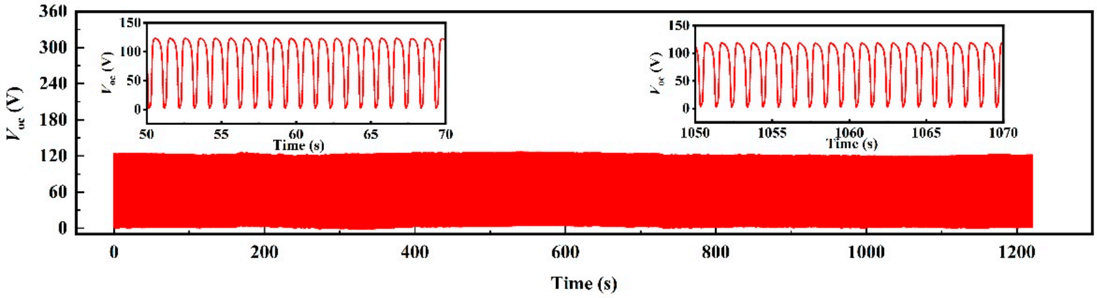


**Figure S6.** Stability curve of PAM-based pressure sensor.

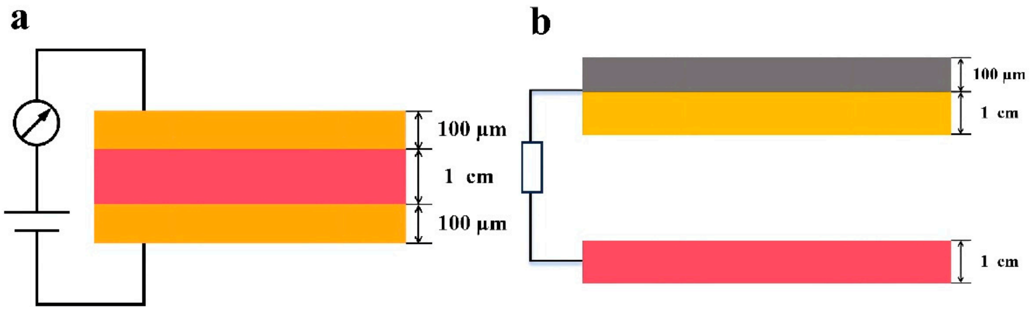


**Figure S7.** Supply electrode thickness and connection mode in (**a**) pressure sensor and (**b**) TENG structural schematics.

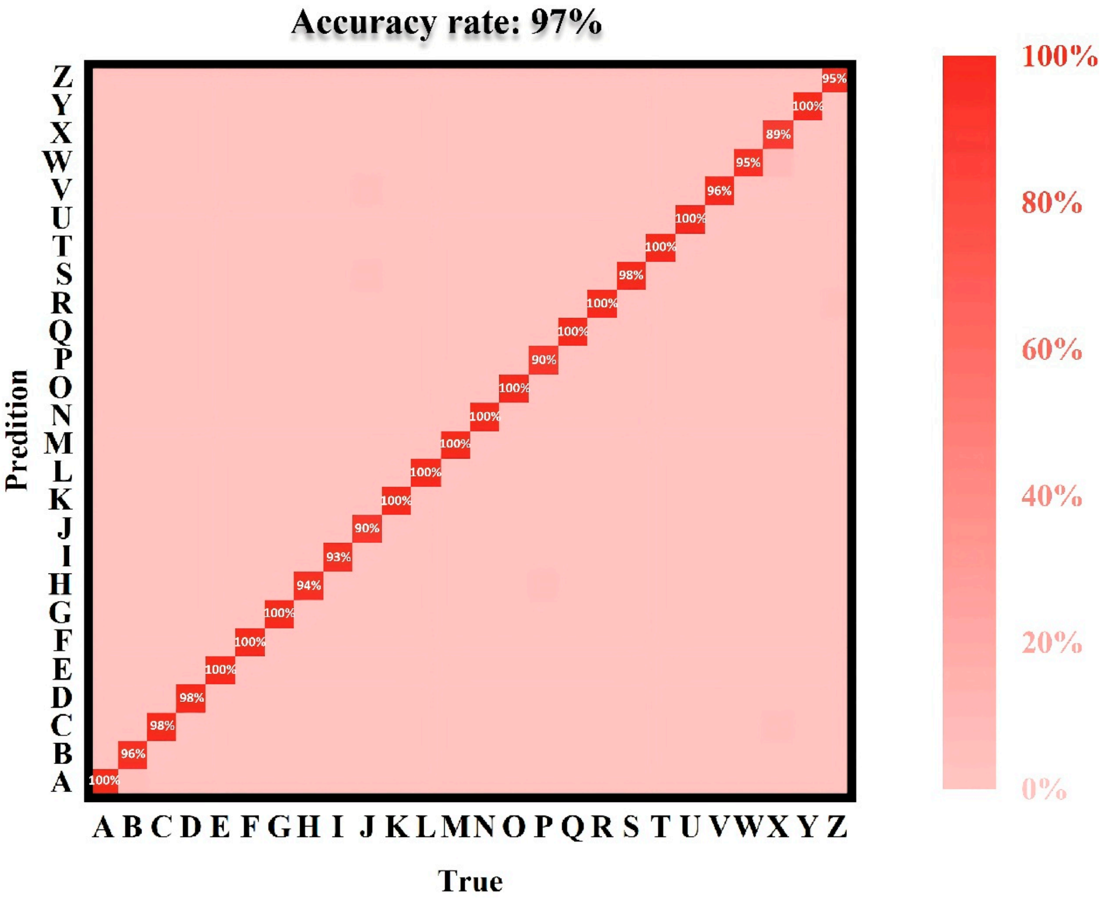


**Figure S8.** Confusion Matrix of MLP-Based Alphabet Recognition with Labeled Precision, Recall and Overall Classification Accuracy.
